# Direct Heme Uptake by Phytoplankton-Associated *Roseobacter* Bacteria

**DOI:** 10.1128/mSystems.00124-16

**Published:** 2017-01-10

**Authors:** Shane L. Hogle, Bianca Brahamsha, Katherine A. Barbeau

**Affiliations:** a Geosciences Research Division, Scripps Institution of Oceanography, La Jolla, California, USA; b Marine Biology Research Division, Scripps Institution of Oceanography, La Jolla, California, USA; Georgia Tech

**Keywords:** roseobacter, diatom, algae, phytoplankton-bacterium interactions, marine, iron transport, heme, iron cycling, remineralization, gene knockout, gene expression

## Abstract

Iron is an essential micronutrient and can limit the growth of both marine phytoplankton and heterotrophic bacterioplankton. In this study, we investigated the molecular basis of heme transport, an organic iron acquisition pathway, in phytoplankton-associated *Roseobacter* bacteria and explored the potential role of bacterial heme uptake in the marine environment. We searched 153 *Roseobacter* genomes and found that nearly half contained putative complete heme transport systems with nearly the same synteny. We also examined a publicly available coculture transcriptome and found that *Roseobacter* strain *Sulfitobacter* sp. strain SA11 strongly downregulated a putative heme transport gene cluster during mutualistic growth with a marine diatom, suggesting that the regulation of heme transport might be influenced by host cues. We generated a mutant of phytoplankton-associated *Roseobacter* strain Ruegeria sp. strain TM1040 by insertionally inactivating its homolog of the TonB-dependent heme transporter *hmuR* and confirmed the role of this gene in the uptake of heme and hemoproteins. We performed competition experiments between iron-limited wild-type and mutant TM1040 strains and found that the wild type maintains a growth advantage when competing with the mutant for iron compounds derived solely from lysed diatom cells. Heme transport systems were largely absent from public marine metagenomes and metatranscriptomes, suggesting that marine bacteria with the potential for heme transport likely have small standing populations in the free-living bacterioplankton. Heme transport is likely a useful strategy for phytoplankton-associated bacteria because it provides direct access to components of the host intracellular iron pool after lysis.

**IMPORTANCE** Ecosystem productivity in large regions of the surface ocean is fueled by iron that has been microbially regenerated from biomass. Currently, the specific microbes and molecules that mediate the transfer of recycled iron between microbial trophic levels remain largely unknown. We characterized a marine bacterial heme transporter and verified its role in acquiring heme, an abundant iron-containing enzyme cofactor. We present evidence that after host cell lysis, phytoplankton-associated bacteria directly extract heme and hemoproteins from algal cellular debris in order to fulfill their iron requirements and that the regulation of this process may be modulated by host cues. Direct heme transport, in contrast to multistep extracellular processing of hemoproteins, may allow certain phytoplankton-associated bacteria to rapidly extract iron from decaying phytoplankton, thus efficiently recycling cellular iron into the wider microbial loop.

## Abstract (precis)

Ecosystem productivity in large regions of the surface ocean is fueled by iron that has been microbially regenerated from biomass. Currently, the specific microbes and molecules that mediate the transfer of recycled iron between microbial trophic levels remain largely unknown. We characterized a marine bacterial heme transporter and verified its role in acquiring heme, an abundant iron-containing enzyme cofactor. We present evidence that after host cell lysis, phytoplankton-associated bacteria directly extract heme and hemoproteins from algal cellular debris in order to fulfill their iron requirements and that the regulation of this process may be modulated by host cues. Direct heme transport, in contrast to multistep extracellular processing of hemoproteins, may allow certain phytoplankton-associated bacteria to rapidly extract iron from decaying phytoplankton, thus efficiently recycling cellular iron into the wider microbial loop.

## INTRODUCTION

Marine phytoplankton are responsible for about half of net global primary productivity, but nearly half of marine primary production is consumed by heterotrophic marine bacteria through the microbial loop (1). Rapidly responding heterotrophic bacteria frequently accompany marine phytoplankton blooms, especially during bloom decline (2), and are important balances to primary productivity (3). These bacteria consume phytoplankton biomass and recycle nutrient elements such as carbon, nitrogen, and iron back into the wider microbial food web. Heterotrophic bacteria require iron to efficiently consume organic carbon because many of the enzymes necessary for bacterial oxidative respiration contain iron as a cofactor. Indeed, heterotrophic marine bacteria can consume a large amount of iron through the microbial loop, and up to 45% of total biological iron uptake in iron-limited waters may be attributed to heterotrophic bacteria (4). Marine bacterial growth efficiency may even be limited by iron availability under certain conditions (5, 6). In the context of phytoplankton bloom decline, iron compounds derived from phytoplankton cells are likely an important nutrient source for heterotrophic bacteria (2).

The marine iron cycle is closely linked to the activities of the ocean microbiome, which is increasingly being recognized as a structured, dynamic, and heterogeneous microscale landscape occupied by organisms employing an enormous diversity of ecological strategies (7). Currently, very little is known about the exact molecular units transferred between biogeochemical iron pools in the marine environment: for example, the iron-containing molecules that marine bacteria consume during the remineralization of lysed or decaying phytoplankton. Recent work has highlighted the role of phytoplankton-bacterium interactions (8) in modulating the marine carbon cycle and has largely focused on mutualistic interactions between heterotrophic bacteria and marine phytoplankton (9, 10). Antagonistic phytoplankton-bacterium interactions are also important in shaping the development of phytoplankton blooms (11, 12), and interactions between marine bacteria and sinking particulate organic matter, much of which consists of living or dead phytoplankton cells, affect the export of carbon to the deep ocean (1). These kinds of microbial interactions likely shape the marine iron cycle as well, but there is currently little experimental data constraining relationships between microbes and specific iron-containing molecules (13).

Here we focus on the *Roseobacter* group as a model heterotrophic bacterial lineage. *Roseobacter* bacteria are among the most abundant *Alphaproteobacteria* groups in the marine environment, are important organic carbon consumers during and after phytoplankton blooms (3), and play important roles in the global biogeochemical cycles of carbon and sulfur (10, 14). Roseobacters are commonly observed living in close association with eukaryotic phytoplankton (8) and can have both mutualistic and antagonistic relationships with their hosts (9, 12, 15). The metabolic diversity of the *Roseobacter* clade indicates that these organisms can utilize multiple organic substrates (16) and are well adapted to dynamic conditions (3). These characteristics suggest that roseobacters play important roles in recycling organic carbon, nitrogen, and phosphorus into the wider microbial microbial food web.

In this study, we sought to explore the role that roseobacters may play in recycling organic iron in the marine environment. Although iron is a critical micronutrient, the microbial processes underlying its biological remineralization remain largely unknown. Bacterial heme uptake is one mechanism that has been hypothesized to recycle particulate organic iron back into the microbial food web (17). Heme is an iron-containing heterocyclic enzyme cofactor, is biologically ubiquitous, and is widespread in the marine environment (18). Prior work has shown that heme comprises approximately 20% of the total iron pool in marine phytoplankton cultures and is present in similar proportions in marine particulate organic matter (19, 20). Because of its prevalence in marine phytoplankton, heme may be a potentially abundant local iron source for bacteria living in close proximity to phytoplankton. Indeed, one prior study identified a *Roseobacter* strain associated with the cyanobacterium *Trichodesmium* that could grow on heme and further identified 19 other *Roseobacter* genomes with putative heme transport gene clusters (21). Due to the lack of a knockout system for the particular strain, that study was not able to conclusively link growth on heme to a specific set of genes or completely distinguish direct heme transport from extracellular heme processing with subsequent free iron transport. Here we expand on those earlier reports of heme uptake in marine roseobacters specifically by confirming the role of the *hmuR* gene in transporting heme and by showing that mutants with an insertionally inactivated *hmuR* gene are deficient in their ability to compete for phytoplankton-derived organic matter under conditions of iron limitation. We further show that *hmuR* is widespread in *Roseobacter* genomes and present evidence that the expression of *Roseobacter* heme transport systems is sensitive to host phytoplankton cues. These results provide insight into the potential role for heme in phytoplankton-bacterium interactions and an additional context for the broader marine biogeochemical cycle of iron.

## RESULTS

### The *Sulfitobacter* sp. strain SA11 heme transporter is downregulated during exponential growth in coculture with a marine diatom.

**FIG 1:**
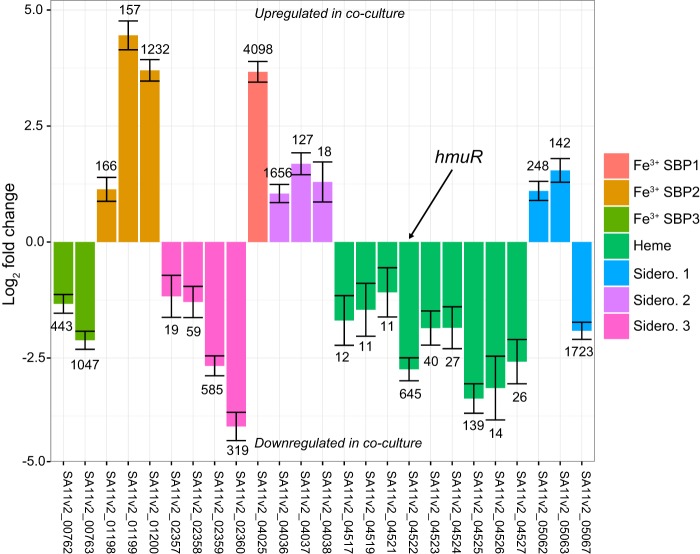
Differential expression of putative *Sulfitobacter* sp. strain SA11 iron transporters between axenic conditions and coculture with the diatom *Pseudo-nitzschia multiseries* PC9. The horizontal axis displays gene locus tags for transcripts with an FDR-corrected *P* value of <0.01 and a log_2_ fold change magnitude greater than 1. The vertical axis displays the log_2_ fold change of SA11 transcripts. Error bars indicate estimated standard errors of log_2_ fold change. The values at the top of the bars represent the variance stabilizing transformed expression levels of each transcript averaged over all samples. Genes with a positive log_2_ fold change were upregulated in coculture, while those with a negative fold change were downregulated. SBP, solute binding protein; Sidero., siderophore utilization gene cluster.

While examining the data from a recent transcriptomic experiment (9), we noticed that a putative heme transport system from a diatom growth-promoting bacterial strain (*Sulfitobacter* sp. strain SA11) was strongly downregulated during symbiotic growth with a marine diatom (*Pseudo-nitzschia multiseries* PC9) compared with axenic bacterial controls. In the original work, transcriptomes were generated from SA11 and *P. multiseries* cocultures harvested at mid-exponential growth (96 h after inoculation). At 96 h in coculture with *P. multiseries*, SA11 upregulated pathways associated with the production of the plant hormone indole-3-acetic acid relative to axenic controls, which stimulated the growth of *P. multiseries*. We examined in this data set the expression of 13 putative iron transport systems encompassing 42 different genes identified in SA11 (see Data Set S1 in the supplemental material). At mid-exponential growth in coculture, there appeared to be a broad shift in the expression of iron transport capabilities in SA11 (Fig. 1). For example, genes encoding a putative heme transporter, two putative siderophore transport systems (“Sid1” and “Sid3”), and a putative ATP-binding cassette transporter (ABCT) system (“Fe^3+^ SBP3”) were significantly downregulated during exponential-growth-phase coculture. For context, the TonB-dependent transporter (TBDT) of the heme transport system was differentially expressed at nearly twice the intensity of the indole-3-acetic acid biosynthesis components reported by Amin et al. (9) (Data Set S1). A different potential siderophore transporter (“Sid2”) and two other putative iron uptake ABCTs (“Fe^3+^ SBP1 and SBP2”) were upregulated during coculture. Most of the genes in the six other potential iron transport systems were either not differentially expressed or had small log_2_-fold changes (see Fig. S1A in the supplemental material). These results suggest that SA11 was differentially regulating multiple potential iron transporters in response to a phytoplankton host, even though iron was not a variable manipulated in the original experiment.

10.1128/mSystems.00124-16.9 DATA SET S1  *Sulfitobacter* sp. strain SA11 processed expression data, iron-related genes identified in the SA11 genome, and expression of indole-3-acetic acid (IAA) and tryptophan biosynthesis pathways identified by Amin et al. (9). Download DATA SET S1, XLSX file, 0.5 MB.. Copyright © 2017 Hogle et al. 2017 Hogle et al. This content is distributed under the terms of the Creative Commons Attribution 4.0 International license.

10.1128/mSystems.00124-16.1 FIG S1  (A) M (log ratio)/A (mean average) (MA) plot generated from a previously published transcriptome sequencing (RNA-seq) experiment investigating interactions between a diatom, i.e., *Pseudo-nitzschia multiseries* PC9, and *Sulfitobacter* sp. strain SA11. Each SA11 gene detected in the RNA-seq experiment is represented as a dot. The horizontal axis displays the variance stabilizing transformed expression of each transcript averaged over all samples (as implemented in the DESeq2 package). The vertical axis displays the log_2_-fold change of SA11 transcripts. Genes with a positive log_2_-fold change were more abundant when SA11 was cocultured with *P. multiseries* (compared with axenic control), while those with a negative fold change were less abundant under coculture conditions. Genes with an FDR-corrected *P* value of >0.05 or a log_2_-fold change magnitude of <1 are shown with 50% opacity. (B) Genomic organization of the *Sulfitobacter* sp. strain SA11 putative heme uptake locus. Genes homologous to the heme uptake locus in TM1040 are colored and labeled equivalently for both (see Fig. S3 and S^4^). Genes colored white are those encoding hypothetical proteins. For reference, the locus name of the putative heme outer membrane receptor is listed underneath *hmuR*, the scaffold name is listed below the diagram, and the base pair position on the scaffold is labeled with a dashed line at each end of the figure. Download FIG S1, TIF file, 0.8 MB.. Copyright © 2017 Hogle et al. 2017 Hogle et al. This content is distributed under the terms of the Creative Commons Attribution 4.0 International license.

### Heme transporters are abundant in marine *Roseobacter* genomes.

**FIG 2:**
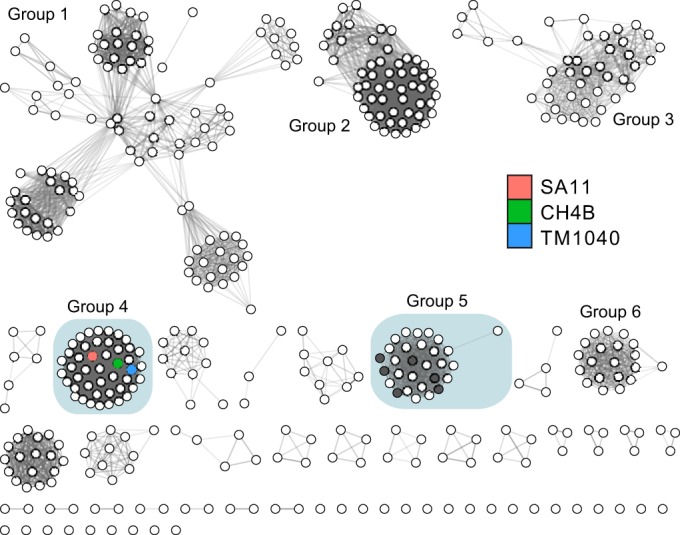
Sequence similarity network of 436 TonB-dependent transporters from 153 different *Roseobacter* genomes. The network is displayed with an E value threshold of 10^−110^ (corresponding to ~34% sequence identity). Increasing edge opacity is proportional to decreasing E value. The 45 sequences in group 4 all have shared synteny with the TM1040 heme transport cluster, and 23 sequences in group 5 are also syntenic (dark gray nodes indicate sequences that lack neighborhood synteny). *Sulfitobacter* sp. strain SA11, *Ruegeria* sp. strain TrichCH4B, and *Ruegeria* sp. strain TM1040 heme TonB-dependent transporters are highlighted for reference.

We explored the distribution of potential heme transporters in the marine *Roseobacter* clade, which is of the same taxonomic lineage as *Sulfitobacter* sp. strain SA11. We examined amino acid homology between the SA11 heme TBDT (Fig. S1B), which is an outer membrane receptor, and 436 other TBDT sequences from 153 different genomes of the *Roseobacter* lineage. We initially identified TBDTs by sequence homology at the protein family level (Pfam accession no. PF00593), but TBDT sequences are divergent, and it is challenging to predict substrate specificity with alignment-based methods alone (22). We instead opted to construct sequence similarity networks (SSNs) as a method for viewing homology between sequences (23). In these network representations (Fig. 2), nodes represent sequences while edges represent similarity scores between sequences. For our TBDT network, edges between nodes are drawn for pairwise similarities corresponding to an E value threshold of 10^−110^, which is equivalent to a mean amino acid sequence identity of 34%. Most *Roseobacter* TBDTs cluster into six groupings of at least 20 sequences each, and the SA11 heme TBDT falls into a distinct cluster with 44 other sequences (group 4), including those of related *Roseobacter* strains *Ruegeria* sp. strain TM1040 and *Ruegeria* sp. strain CH4B. All TBDTs in group 4 share a syntenic gene neighborhood (10 genes upstream or downstream) that includes other genes predicted to be involved in heme transport (24). Additionally, most of the TBDT sequences (with the exception of six sequences) in group 5 have synteny similar to that of the SA11 heme transport system, although they are distinct enough to fall into a separate cluster. Generally, the genome neighborhoods of the nonheme TBDT clusters are much less regularly ordered than those seen with group 4 and group 5. Based on gene cluster synteny and sequence clustering, it appears that 16% of all *Roseobacter* TBDT genes code for heme transporters. As a result, 45% of the 153 *Roseobacter* genomes surveyed here appear to have a complete heme transport system.

### The TM1040 genome contains a putative heme transporter.

*Ruegeria* sp. strain TM1040 is a genetically tractable *Roseobacter* strain, which makes it an appealing target for physiological and genetic investigations. The putative heme transport clusters in both SA11 (Fig. S1B) and TM1040 (Fig. S2) are syntenic. The heme transport TBDTs from these clusters share 48% amino acid identity. The TM1040 genome contains 10 colocalized genes (Fig. S2) organized similarly to heme transport clusters in other Gram-negative bacteria (24). The 10 genes are organized in two divergently transcribed operon-like structures and include putative components of sequences encoding an ATP binding cassette transporter (ABCT) system (*hmuTUV*), a heme oxygenase (*hmuS*), the outer membrane TBDT (*hmuR*), and the energy transduction components of the TBDT system (*exbB*, *exbD1*, *tonB*). A duplicate of the *exbD1* gene (*exbD1a*) and a gene encoding a hypothetical protein are also included in the putative TM1040 heme transport operons. The top BLAST hit for the TM1040 *hmuR* gene (UniProtKB accession no. Q1GJT6) in the curated Swiss-Prot database (25) is to *Haemophilus ducreyi* (23.6% amino acid identity; UniProtKB accession no. Q7VNU1), while the top hit of TM1040 *hmuS* is to *Yersinia pestis* (36.8% amino acid identity; UniProtKB accession no. Q56990).

10.1128/mSystems.00124-16.2 FIG S2  TM1040 heme transporter diagram and characterization of cotranscribed genes. The top diagram displays TM1040 heme transport genes, the scale bar equals a 1-kb pair, arrows represent primers used in RT-PCR, and lines denote the PCR products predicted for each primer pair and their lengths and orientation. The lower diagram shows the results of the RT-PCR cotranscription analysis. Each primer pair shown at the top produced one or more bands after PCR amplification. The band consistent with the expected product length (shown at the top) is highlighted with an arrowhead in the cases where multiple bands are observed. Primers are listed by their shorthand names (see Table S4). The first and last lanes contain a 1 Kb Plus DNA Ladder (Invitrogen). The second lane represents a negative control containing mRNA divided into aliquots prior to reverse transcription indicating no contaminating genomic DNA. Download FIG S2, TIF file, 1.3 MB.. Copyright © 2017 Hogle et al. 2017 Hogle et al. This content is distributed under the terms of the Creative Commons Attribution 4.0 International license.

### The TM1040 heme transporter is upregulated under iron stress conditions and cotranscribed.

We examined the expression of the 10 putative heme transport genes under iron-limiting (no added iron) and iron-replete (5 μM FeCl_3_ added) conditions using two-step reverse transcription-quantitative PCR (RT-qPCR). All genes in the TM1040 putative heme transport operons were upregulated between approximately 41-fold and 201-fold under iron stress conditions relative to iron-replete conditions (Fig. S3). The three most highly expressed genes were those encoding the putative TBDT (*hmuR*), the solute binding protein of the ABCT system (*hmuT*), and the hypothetical protein (*hyp*). The gene encoding the putative heme oxygenase (*hmuS*), which has been used as a diagnostic gene for identifying heme transport systems (18), was also upregulated more than 140-fold. We also analyzed cotranscription of consecutive genes in the putative heme transport gene clusters as has been described previously (26). Although the 10 components were not upregulated at uniform levels under iron stress conditions, the genes in each divergent cluster were cotranscribed. RT-PCR amplification products spanning junctions of the six genes in the *hmuR* cluster and four genes in the *hmuS* cluster were observed in all cases (Fig. S2), indicating cotranscription on the respective strands.

10.1128/mSystems.00124-16.3 FIG S3  RT-qPCR of expression changes for the TM1040 heme uptake locus under iron-limited compared with iron-replete culture conditions. Relative expression is displayed as an expression ratio (*n* = 3 biological replicates per group) normalized against three condition-stable reference genes—*rpoD*, *gyrA*, and *gmkA*. Ratios greater than 1 indicate higher gene expression in iron-stressed cultures. Asterisks indicate genes determined to be differentially expressed (*P* < 0.05). Download FIG S3, TIF file, 0.2 MB.. Copyright © 2017 Hogle et al. 2017 Hogle et al. This content is distributed under the terms of the Creative Commons Attribution 4.0 International license.

### TM1040 can utilize heme or hemoglobin as an iron source.

**FIG 3:**
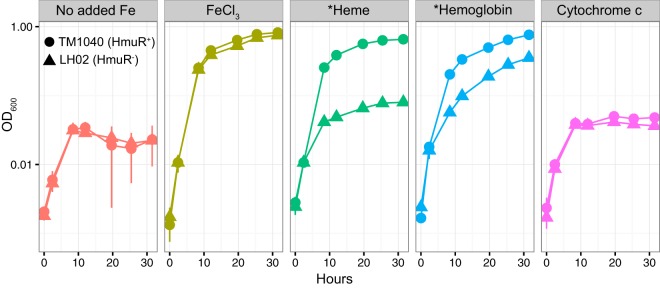
Growth of wild-type TM1040 (HmuR^+^) and LH02 (HmuR^−^) strains with different added iron sources. The vertical axis (log_10_ scale) displays optical density measurements at 600 nm (OD_600_) with respect to time. Conditions indicated with an asterisk had statistically different growth rates between LH02 and TM1040 (independent, two-tailed Student’s *t* test, *P* < 0.05). All iron sources were added at 500 nM except for hemoglobin, which was added at 17 μg/ml.

Because the TM1040 genome contains a putative heme transport system that was strongly upregulated under iron stress conditions, we examined the growth of TM1040 on inorganic FeCl_3_, hemin chloride (heme), and the model hemoproteins hemoglobin (Hb) and cytochrome *c* (cyt *c*). The specific growth rate of TM1040 in an iron-depleted medium with a 500 nM concentration of added inorganic FeCl_3_ was 0.31 ± 0.02 h^−1^, while it was significantly lower (0.13 ± 0.004 h^−1^) in the absence of a supplemented iron source. TM1040 grew at a level comparable to that seen under FeCl_3_ conditions when we provided 500 nM heme as the iron source (0.28 ± 0.01 h^−1^). We then supplied TM1040 with 17 μg/ml Hb and 500 nM cyt *c* in order to determine whether TM1040 could utilize more structurally complex hemoproteins. In cyt *c*, the heme prosthetic group is covalently bound to the parent protein while heme is noncovalently embedded in the Hb tetramer. TM1040 was able to grow as well with Hb (0.28 ± 0.01 h^−1^) as with FeCl_3_ but not cyt *c* (0.14 ± 0.01 h^−1^), suggesting that the structural nature of the parent protein may be an important factor in determining the bioavailability of heme (Fig. 3; see also Table S1 in the supplemental material). Covalently linked hemoproteins may require additional enzymatic processing or chemical decomposition before they are rendered bioavailable, although it is possible that other, yet-to-be-identified bacterial strains possess the enzymatic capacity to use them directly.

10.1128/mSystems.00124-16.5 TABLE S1  Growth rates of TM1040 and LH02 on different iron sources. *P* values indicate the results of an independent two-tailed Student’s *t* test comparing the strain growth rates under each condition. Values greater than 0.05 are omitted. There were three replicates for each condition. Download TABLE S1, DOCX file, 0.05 MB.. Copyright © 2017 Hogle et al. 2017 Hogle et al. This content is distributed under the terms of the Creative Commons Attribution 4.0 International license.

### Disruption of *hmuR* eliminates TM1040 growth on heme.

We selected the TM1040 *hmuR* gene for insertional inactivation in order to determine how this particular gene was involved in TM1040’s observed growth on heme and human Hb. We targeted *hmuR* because it was the most dramatically upregulated gene in the heme transport cluster under iron stress conditions and also encodes a putative outer membrane TBDT, which is the first step in the transfer of heme from the extracellular environment into the bacterial cytoplasm. Polar effects from the disruption of *hmuR* could potentially affect the expression of downstream genes for the energy transduction components of the heme transport cluster but most likely would leave the inner membrane transport machinery in the divergent gene cluster unaffected. Therefore, any indirect effects from the inactivation of *hmuR* would limit disruption to genes involved in energy transduction and substrate movement across the bacterial outer membrane. We deleted a 435-bp portion of the TM1040 *hmuR* gene beginning at position 975 and replaced the deletion with a neomycin phosphotransferase II gene conferring kanamycin resistance (resulting in the recombinant strain Δ*hmuR*975::*nptII*). The resulting HmuR^−^ mutant strain was named LH02 (Table S2 and Fig. S4). TM1040 and LH02 grew equally well when supplied with FeCl_3_ as an iron source, but LH02 growth on heme and hemoglobin was greatly reduced (Fig. 3 and Table S1), indicating that the *hmuR* gene is necessary for growth on heme.

10.1128/mSystems.00124-16.6 TABLE S2  Bacterial strains and plasmids used in this work. Download TABLE S2, DOCX file, 0.1 MB.. Copyright © 2017 Hogle et al. 2017 Hogle et al. This content is distributed under the terms of the Creative Commons Attribution 4.0 International license.

10.1128/mSystems.00124-16.4 FIG S4  (A) pLH02 plasmid generation from two Gibson assembly steps. The first step (top green horizontal line) depicts production of plasmid pLH01 using a Gibson assembly kit (New England BioLabs). The second step (bottom green horizontal line) depicts the final insertion of the kanamycin-resistant cassette between upstream (blue) and downstream (pink) regions of the TM1040 *hmuR* gene again. Red arrowheads denote positions of primers (Table S^4^) used to produce DNA fragments that were later assembled by Gibson reaction. (B) PCR verification of TM1040 strain LH02. (Right) Genomic organization of *hmuR* and surrounding genes in the TM1040 wild type (WT) (top) and TM1040 LH02 (bottom). Red arrows denote primers (Table S^4^) used to verify the double-crossover event in LH02. In LH02, the upstream region from the original *hmuR* gene is shown in blue and the downstream region is shown in pink. (Left) Gel showing PCR products obtained using primers insert_cnfrm_fwd and insert_cnfrm_rev on TM1040 WT (left lane) and TM1040 LH02 (right lane). The first lane contains a 1 Kb Plus DNA Ladder (Invitrogen). Download FIG S4, TIF file, 1.9 MB.. Copyright © 2017 Hogle et al. 2017 Hogle et al. This content is distributed under the terms of the Creative Commons Attribution 4.0 International license.

### TM1040 outperforms LH02 when diatom lysate is the only added iron source.

**FIG 4:**
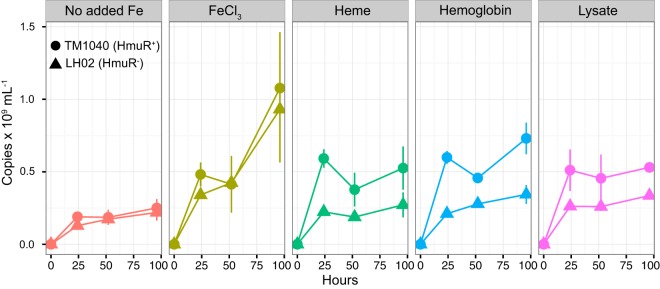
Competition between cocultured TM1040 (HmuR^+^) and LH02 (HmuR^−^) for different iron sources. The abundance of each strain was determined by qPCR using primers targeting either the kanamycin resistance cassette for LH02 or the region of the *hmuR* gene deleted in LH02 and retained in TM1040. The vertical axis displays the absolute concentration of qPCR copies detected from each strain (×10^9^ ml^−1^) with respect to time. FeCl_3_ and heme were added at 500 nM, hemoglobin was added at ~17 μg/ml, and algal lysate was added at a concentration providing ~11 nM heme equivalents.

In order to simulate natural conditions where TM1040 cells would be in contact with heterogeneous dissolved and particulate organic matter, we tested whether TM1040 was able to outperform LH02 when soluble and insoluble fractions of lysed diatom cells were supplied as the added iron source. Specifically, we tested whether TM1040 or LH02 would numerically outcompete the other when cocultured with 500 nM heme, 17 μg/ml Hb, 500 nM FeCl_3_, or cellular lysate obtained from the centric marine diatom *Thalassiosira pseudonana*. Axenic *T. pseudonana* lysate was prepared by first removing extracellular iron from harvested algal cells in order to prevent confounding effects from extracellular inorganic iron species (27). Using high-performance liquid chromatography (HPLC), we determined the heme content of the original axenic *T. pseudonana* cultures to be approximately 0.72 ± 0.09 nM with a chlorophyll *a*/heme *b* ratio of 264 ± 38, which is in good agreement with heme *b* measurements from other marine phytoplankton (Table S3) (28). After determining the heme concentrations in the lysates, we added *T. pseudonana* lysate to cocultures under conditions that were strictly free of trace metals (trace metal clean) (see Materials and Methods) in order to generate a concentration of approximately 11 nM heme equivalents. To quantify strain proportions in coculture, we developed a qPCR assay with primers targeting the inserted *nptII* gene in LH02 and the corresponding 435-bp portion of the TM1040 *hmuR* gene that was deleted in LH02. TM1040 outcompeted LH02 under all conditions, except for coculture with 500 nM FeCl_3_ or in the absence of additional iron (Fig. 4). Coculture with FeCl_3_ resulted in the highest number of amplicon copies per milliliter, while the no-iron-addition conditions resulted in the lowest. We observed the greatest difference in strain proportions when TM1040 and LH02 were cocultured with heme and hemoglobin. When grown on *T. pseudonana* lysate, the wild-type strain outcompeted LH02 in roughly similar proportions in growth with heme and hemoglobin but the lysate supported a lower number of amplicon copies per milliliter than heme or hemoglobin.

10.1128/mSystems.00124-16.7 TABLE S3  Measurements of heme content in soluble and insoluble *T. pseudonana* lysate. A superscript italic a indicates the cell concentration before the washing and concentration steps; the data were used to calculate the chlorophyll *a* (Chl a) level per cell. A superscript italic b indicates the cell concentration after the washing and concentration steps; the data were used to calculate the heme *b* level per cell. Download TABLE S3, DOCX file, 0.05 MB.. Copyright © 2017 Hogle et al. 2017 Hogle et al. This content is distributed under the terms of the Creative Commons Attribution 4.0 International license.

## DISCUSSION

*Roseobacter* is an abundant lineage of marine heterotrophic bacteria that play important roles in the biogeochemical cycling of major nutrient elements (10, 14). For some particular roseobacters, such as *Ruegeria* sp. strain TM1040 and *Sulfitobacter* sp. strain SA11, heme transport appears to be an important mode of iron acquisition. TM1040 was originally isolated from the bloom-forming dinoflagellate *Pfiesteria piscicida* (29, 30), is motile, and degrades alga-produced dimethylsulfoniopropionate (DMSP) (31). SA11 was isolated from the marine diatom *Pseudo-nitzschia multiseries* PC9 and has been shown to be important in promoting the growth of its algal partner via production of a hormone (9). Other members of the *Roseobacter* clade have frequently been isolated or detected in association with phytoplankton and other biological surfaces (32) and have been documented to increase in abundance during algal blooms (33). The close association of many *Roseobacter* strains with algal cells (3) and the abundance and conservation of synteny of putative heme transport genes within the clade suggest that nearly half of 153 genome-sequenced members likely utilize heme as an iron source, but this estimate is likely biased against important but underrepresented genome-streamlined roseobacters (34, 35). Heme transport systems may be a common feature of larger-genome, phytoplankton-associated roseobacters.

Our results strongly suggest that *Ruegeria* sp. strain TM1040 acquires heme and certain hemoproteins by direct transport through the HmuR outer membrane TBDT, in contrast to a multistep pathway where iron is released from the heme molecule extracellularly prior to transport. The TM1040 genome contains only a single outer membrane TBDT and eight potential outer membrane porins. Gram-negative bacterial heme transport has been shown to occur only through outer membrane TBDTs (24), thus strongly suggesting that the only viable pathway for direct heme transport in TM1040 is through its single outer membrane TBDT. The greatly reduced growth of HmuR^−^ mutant LH02 compared with TM1040 when supplied with heme and hemoproteins indicates that the growth difference is caused by a differential capacity for the two strains to acquire heme, likely through direct transport by HmuR. TM1040 could also possibly release iron from heme and hemoproteins extracellularly and obtain the resulting free iron through a transporter other than HmuR, but this two-step strategy would have to be much less efficient; otherwise, we would have observed a much smaller difference between LH02 and TM1040 in growth results. Our results do not preclude the possibility of an alternative heme transport pathway in TM1040, but they do suggest that HmuR was by far the most efficient outer membrane heme transporter under the experimental conditions tested here.

LH02 growth on heme was greatly diminished, but LH02 was still able to persist during the stationary-growth phase when competing with TM1040 (Fig. 4) and when grown alone (Fig. 3). The growth medium used for these experiments was based on natural coastal seawater, and although we purified the medium by removing trace metals, there was certainly residual iron that persisted after our preparations. We suspect that LH02 was not eliminated from experiments where heme or hemoproteins were provided as added iron sources because there was sufficient residual iron in the background medium to support small populations of mutant cells. This iron likely existed in many different organic and inorganic forms and may have been rendered bioavailable by additional extracellular processing and/or due to kinetic dissociation to bioavailable forms. The presence of residual iron derived from the background medium as well as from the phytoplankton lysate likely also explains the persistence of LH02 during competition experiments. Phytoplankton cells contain many different intracellular forms of iron, including heme, iron-sulfur clusters, and mononuclear nonheme forms. All of these chemical forms were undoubtedly present in the diatom lysate that we added in the competition experiments, and nonheme forms were presumably equally bioavailable to LH02 and TM1040. Potentially, LH02 was able to subsist on nonheme iron and was therefore not driven to extinction in these experiments.

There is also a possibility that the disruption of the *hmuR* gene produced a fitness disadvantage for LH02 that was unrelated to the transport of heme or other iron-containing molecules. In some experiments, we did notice a small reduction in LH02 culture density compared with that of TM1040 (Fig. 3) when the two strains were equally iron replete or iron limited. However, the growth rates of the two were not statistically different in either case (see Table S1 in the supplemental material). We speculate that this small difference may have been due to the internal recycling of heme from dying cells when wild-type cultures reached or approached stationary phase. We also noticed a small divergence between the wild type and the mutant in iron-replete and iron-limited controls in the competition experiments (Fig. 4), although this divergence was within the error bars of biological replicates. Despite this small divergence in the controls, the heme, hemoglobin, and diatom lysate produced much larger differences between LH02 and TM1040, suggesting that it was heme or hemoprotein availability that was primarily driving the observed differences.

We were curious to explore the environmental distribution of heme transporters in natural marine microbial communities. Heme transport systems were reported to be scarce in earlier experiments using marine metagenomic data sets (36, 37), but to our knowledge, no searches of contemporary data sets had yet been performed. As a part of this study, we performed an updated search of publically available metatranscriptomes and metagenomes, including global ocean sampling (GOS), Tara Oceans, and the data set used by Teeling et al. (33), but these searches yielded few positive hits. However, most current public metagenomes and metatranscriptomes have disproportionately targeted the small, free-living fraction of marine bacterioplankton, and genomes of the major constituents of this fraction, including *Pelagibacter*, *Prochlorococcus*, *Synechococcus*, SUP05, SAR116, and SAR86, lack heme transporters. This suggests that marine bacteria with the potential for heme transport comprise a relatively small fraction of planktonic bacterial communities, potentially due to strong grazing pressure and/or planktonic dormancy. However, bacteria that are typically rare in metagenomes but can be transiently abundant are increasingly being recognized as disproportionate contributors to marine carbon recycling (38) and ecosystem-wide gene transcription (39). These same strains may disproportionately affect the marine iron cycle as well. We hypothesize that marine bacterial heme transport systems may be better represented in larger fractions (>5 μm), particularly during the decline and senescence of phytoplankton blooms.

Previous work suggested that heme uptake is a mechanism by which heterotrophic bacteria remineralize particulate organic iron in the marine environment (17, 21). Phytoplankton cells contain relatively high levels of hemoproteins that are mostly associated with the photosynthetic apparatus (40), and lysed or decaying phytoplankton cells could be a significant source of heme or hemoproteins for nearby bacteria. In a prior study in terrestrial systems, a heme transporter was genetically characterized in the *Alphaproteobacteria* N_2_-fixing plant symbiont *Bradyrhizobium japonicum* (41). The authors hypothesized that this system would be advantageous for *B. japonicum* when the plant root nodule that harbored the bacterium lost integrity and began to decay and the bacterium was forced to adapt to a new free-living lifestyle where leghemoglobin, a hemoprotein found in the nitrogen-fixing root nodules of leguminous plants, was a new and abundant iron source (41). It seems likely that a similar strategy would be appropriate in a marine context, particularly that of heterotrophic bacteria interacting with phytoplankton and other marine biogenic particles. Indeed, the results from experiments analyzing competition between TM1040 and LH02 suggest that the heme transport system provides an advantage to TM1040 when these strains are competing for biogenic iron derived from algal material (Fig. 4), which in turn suggests that hemoproteins derived from algal material are iron sources for TM1040 in the wild. Furthermore, our findings from the transcriptomic experiment performed with SA11 suggest that the regulation of heme transport is sensitive to coculture with an algal host (Fig. 1). Although this observation is not mechanistically conclusive, it does suggest that heme transport may not be an advantageous strategy when host and symbiont are growing symbiotically. This is consistent with our findings showing that TM1040 can use hemoproteins derived from lysed and decaying phytoplankton, suggesting that heme compounds likely become relevant iron sources upon host death. Based on transcriptomic evidence, it appears that SA11 shifted its iron uptake strategy away from the use of organic iron resources (heme and two potential siderophores) and toward uptake of small inorganic iron(III) chemical species during coculture.

**FIG 5:**
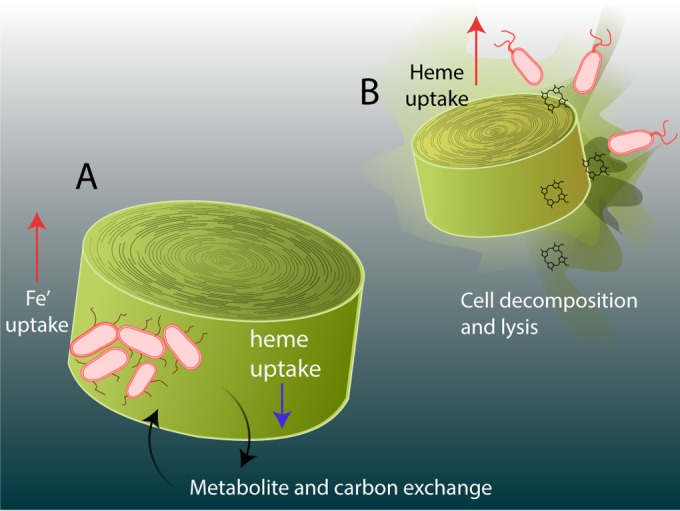
Model of *Roseobacter* heme utilization and algal host growth. (A) During exponential growth, attached bacteria and the algal cell exchange carbon and metabolites in a mutualistic relationship. Bacteria shift their iron acquisition strategy away from heme (blue arrow, downregulation) uptake and toward other “free” iron species (red arrow, upregulation). (B) During algal cell decomposition, bacterial cells potentially transition to a motile growth phase in order to search out a new host while simultaneously targeting newly abundant heme and hemoproteins as iron resources (red arrow, upregulation).

TM1040 has been observed to oscillate between mutualistic and antagonistic lifestyles depending on the concentrations of algal lignin decomposition by-products (15). In the first stages of phytoplankton-bacterium interaction, motile TM1040 colonizes the phytoplankton cell surface and enters a sessile life phase where it utilizes phytoplankton-derived DMSP and other carbon compounds while simultaneously producing a suite of small molecules from which the host benefits. Algal senescence triggers the biosynthesis in TM1040 of a small infochemical that stimulates TM1040 motility and also stimulates algal lysis. As the host cell lyses and dies, it is plausible that algal hemoproteins become a relatively abundant iron resource for the newly motile TM1040 as it searches out a new algal symbiont. We propose an additional iron component to the “swim or stick” model (15, 42) where *Roseobacter* symbionts utilize different suites of iron compounds based on whether they are growing in association with a living algal cell or are transitioning to a new lifestyle when their algal host dies (Fig. 5). In particular, heme transport is downregulated during bacterium-alga symbiosis, presumably due to a lack of availability of heme-containing molecules, while it is upregulated during cell decomposition and becomes an advantageous mode of iron nutrition for the bacteria as they are transitioning to a new host cell.

### Conclusion.

Regenerated iron is estimated to comprise between 50% (43) and 90% (44) of the total iron supply in marine planktonic ecosystems, and it is estimated that nearly 25% of all particulate iron in some ocean surface waters is routed through heterotrophic bacteria (45). More recently, heme in marine particulate organic matter has been directly measured at picomolar concentrations (19, 20). Few studies have examined turnover rates of algal iron in the marine environment, but if we assume an average contribution of 35 pM diatom-derived particulate organic iron during natural iron-fertilized diatom blooms (13, 43) and a mobilization rate of 5% to 17% particulate algal iron day^−1^ into heterotrophic bacteria biomass (45) and that 40% of diatom iron is in the form of heme (28), this would translate to a maximum rate of between 0.7 and 2.4 pmol diatom heme liter^−1^·day^−1^ transferred into heterotrophic bacteria during bloom remineralization. If algal heme remains structurally unaltered during bacterial remobilization and direct heme transport is solely responsible for remobilization, we estimate that direct heme transport could in theory account for between 7% and 24% of the total standing heterotrophic bacterial iron inventory during diatom blooms (13, 43). Although heme transport systems appear to be rare in the free-living bacterioplankton, direct heme uptake may be a useful iron acquisition strategy for phytoplankton-associated bacteria, particularly those in the *Roseobacter* clade. Under dense phytoplankton bloom conditions, direct heme transport by heterotrophic bacteria could be a significant mechanism by which particulate organic iron is recycled into the microbial loop.

## MATERIALS AND METHODS

### Transcriptome data from *Sulfitobacter* sp. strain SA11 in coculture with *Pseudo-nitzschia multiseries* PC9.

Transcriptome data were downloaded from the NCBI Gene Expression Omnibus (GEO GenBank accession number GSE65189) (46). To identify genes that were differentially expressed under coculture and axenic culture conditions, SA11 count data were processed using the DESeq2 (47) package in R. The DESeq2 procedure was performed with default parameters using the conventional null hypothesis of zero logarithmic fold change between conditions and a false-discovery-rate (FDR) threshold of 10% (*P*_fdr corrected_ = <0.1). Iron transporters were annotated using The NCBI Conserved Domain Database (48) and cutoffs as described earlier (49), and the resultant data are available in Data Set S1 in the supplemental material.

### Construction of TonB-dependent receptor sequence similarity network.

The genomes of 153 different *Roseobacter* strains from the IMG database (50) were searched for the TBDT domain (Pfam accession no. PF00593). A total of 436 *Roseobacter* TBDT sequences were compared pairwise using BLAST v 2.2.3, and BLAST output was visualized as a sequence similarity network (SSN) using cytoscape (v 3.2.0). SSNs do not rely on a global sequence alignment but rather visualize individual pairwise relationships between sequences (23). An E value threshold of 10^−110^ (corresponding to a mean of 34.3% identity) was chosen for network visualization by manual iteration of increasingly stringent thresholds until clusters persisted through three subsequent iterations (23, 51). Although sequence identity is useful in aiding annotation, TBDTs are most commonly functionally assigned based on the predicted functions of neighboring genes (22, 49). In order to assist the functional annotation of TBDT SSN clusters, the resulting SSN was submitted to the Genome Neighborhood Network tool at the Enzyme Function Initiative (52) with a gene neighborhood size of 10 and a co-occurrence lower limit of 20%, resulting in a collection of genes encoding Pfam proteins present in the genome neighborhood (±10 genes) of at least 20% of the TBDT sequences in each sequence similarity network cluster.

### Bacterial/algal strains and growth conditions.

*Escherichia coli* DH5α (New England BioLabs) was grown in LB medium, and the antibiotics kanamycin (50 μg/ml) and chloramphenicol (10 μg/ml) were used for the selection and maintenance of plasmids. *Ruegeria* sp. strain TM1040 was grown in either marine broth (MB) 2216/marine agar 2216 (Difco) or a heart infusion seawater (HISW)-based medium (25 g heart infusion broth [Difco], 15 g sea salts [Sigma], 1.5% agarose) at either 22°C or 30°C. Kanamycin (120 μg/ml) and chloramphenicol (10 μg/ml) were used for selection in plates and liquid cultures to ensure selection of insertion mutants. Iron-limited cultures were grown in a modified PC medium (21) here termed PC+ medium. Briefly, the medium consisted of 1 liter of 0.2 μM filtered north Pacific seawater collected from the Scripps Institution of Oceanography pier, 1 g bacteriological peptone, 1 g casein, 10 mM glucose, 4.7 mM NH_4_Cl, 600 μM KH_2_PO_4_, 50 μM Na_2_-EDTA, 40 nM ZnSO_4_, 230 nM MnCl_2_, 25 nM CoCl_2_, 10 nM CuSO_4_, 100 nM Na_2_MoO_4_, and 10 nM Na_2_SeO_3_. All components except for the trace metals, KH_2_PO_4_, and EDTA were mixed, filtered through a 0.2-μm-pore-size filter, stirred for 24 h with 7% (wt/vol) Chelex resin (Bio-Rad), and then filtered through a 0.2-μm-pore-size filter to remove the Chelex resin. Trace metal stocks were prepared in 0.1 M HCl and combined in the proportions necessary to generate a mastermix. KH_2_PO_4_ and EDTA were prepared in Milli-Q (MQ) water. Stocks were filter sterilized (using a 0.2-μm-pore-size filter) before use, and KH_2_PO_4_, EDTA, trace metal mastermix, and FeCl_3_ were added to PC+ aliquots immediately before inoculation with TM1040. FeCl_3_ was added to generate final concentrations of 50 nM, 100 nM, 500 nM, and 5 μM.

Cultures of *Thalassiosira pseudonana* were made axenic by transferring the cultures three times in a 1:10,000 dilution through F/2-enriched seawater medium with 100 μg/ml ampicillin, 25 μg/ml streptomycin, and 25 μg/ml neomycin. To test for bacterial contamination, 200-μl aliquots of *T. pseudonana* were plated on 2216 marine agar, incubated for 48 h at 30°C in the dark, and examined visually for bacterial growth.

### Culturing strains under iron-depleted conditions.

Bacterial colonies were inoculated into 5 ml of marine broth (MB) 2216 or HISW medium and allowed to grow at 30°C in the dark for 12 h on a platform shaker (190 rpm). A 250-μl volume of this culture was then transferred into PC+ medium with 5 μM FeCl_3_ or no added iron and grown at room temperature in the dark for 12 h. Then, 250 μl of TM1040 in PC+ medium with 5 μM FeCl_3_ was transferred to a fresh 5-ml volume of PC+ medium with 5 μM FeCl_3_ and allowed to grow for 12 h at room temperature. The same procedure was performed for the culture with no added iron. Iron limitation was confirmed by spiking 5 μM of FeCl_3_ into the iron-depleted culture and observing renewed growth. Fresh stocks of heme were prepared immediately before each experiment by dissolving the required amounts of hemin chloride (Sigma) in 0.3 M ammonium hydroxide. The pH of the solution was adjusted to 8.0 with concentrated HCl, and then the solution was filter sterilized through a 0.2-μm-pore-size membrane. Dissolved heme prepared in this fashion has been determined to have an upper limit of free iron of approximately 4 nM per 1 μM of hemin chloride (53). Stocks of hemoproteins were freshly prepared before each experiment by dissolving the required mass of protein in Milli-Q water and filter sterilizing through a 0.22-μm-pore-size membrane. Significant contamination of free iron in growth media was observed by the use of lyophilized hemoglobin from commercial sources, confounding the growth effect differences between mutant LH02 and wild-type TM1040. To circumvent this contamination, purified human hemoglobin was prepared in the Skaar laboratory at Vanderbilt University by anion exchange high-performance liquid chromatography and protein dialysis (54) and used in all subsequent experiments. All experiments were conducted in the dark to prevent photodegradation of heme and hemoproteins.

### TM1040 genomic DNA extraction.

A 1.5-ml volume of TM1040 culture at an optical density at 600 nm (OD_600_) of approximately 0.6 in MB 2216 medium was pelleted by centrifugation. DNA was extracted from cell pellets using a DNeasy blood and tissue kit (Qiagen) following the manufacturer’s protocols. DNA was quantified using a NanoDrop 1000-D Spectrophotometer (NanoDrop Technologies).

### TM1040 RNA extraction.

TM1040 cultures were grown for 12 h in PC+ medium supplemented with 5 μM of FeCl_3_ or without added iron. Cultures were grown in triplicate. After 12 h, iron-depleted cultures had an average OD_600_ of 0.045 and iron-rich cultures had an OD_600_ of 0.22. A 5-ml volume of triplicate iron-depleted and iron-rich cultures was spun at 4,000 rpm for 10 min at 4°C. The resulting pellets were resuspended in 1 ml TRI-Reagent (Zymo Research), and total RNA was extracted using a Direct-zol RNA MiniPrep kit (Zymo Research) following the manufacturer’s instructions. Traces of contaminating genomic DNA were removed from total RNA using Turbo DNase (Life Technologies, Inc.) following the manufacturer’s instructions. Total RNA concentrations were measured using a NanoDrop 1000-D Spectrophotometer. The absence of contaminating DNA was confirmed by visually inspecting (1% agarose gel) PCRs using the primers for *rpoD* (rpoD_F and rpoD_R) and the total RNA.

### Reverse transcription of mRNA.

Between 0.3 and 1 μg of total extracted RNA was reverse transcribed using random hexamer primers and SuperScript III First-Strand Synthesis SuperMix (Invitrogen) following the manufacturer’s protocol. Resulting cDNA concentrations were measured using a NanoDrop 1000-D Spectrophotometer, and samples were diluted to a final concentration of 5 ng/μl for use in reverse transcription-quantitative PCR (RT-qPCR).

### Reverse transcription-quantitative PCR (RT-qPCR).

RT-qPCR reactions were carried out on a Qiagen RotorGene-Q (Qiagen) using Promega GoTaq qPCR Mastermix in 25-μl total reaction volumes. The diluted cDNA samples (three biological replicates for each of the two iron conditions) were run in triplicate as technical replicates. Quantitative PCRs using total purified RNA were run in triplicate for the biological replicates of each iron condition for the genes *rpoD* and *hmuR* (with primer pair rpoD_F and rpoD_R and primer pair hmuR_F and hmuR_R). Results were below the detection limit and never higher than the cycle threshold values for the no-template controls, demonstrating the absence of genomic DNA contamination in samples. Five-point standard curves ranging from 311,268 to 31 genome copies per 25 μl of reaction mixture in 10-fold dilutions were generated for each gene of interest using TM1040 genomic DNA with the formula *m* = (*n*)1.096 × 10^−21^, where *m* is the genome mass and *n* is the size of the genome in base pairs. For each individual calibration curve, amplification efficiency as calculated from the slope of the standard curves was always greater than 90% and less than 100%. Cycle threshold values for standard curves were used with REST 2009 software (55) to generate expression values for each gene relative to iron-depleted conditions and normalized to the housekeeping genes *rpoD*, *gyrA*, and *gmkA*. The significance of expression ratio values was calculated using the native randomization and bootstrapping algorithms in REST 2009 software.

### Partial deletion and insertional inactivation of *hmuR* in the TM1040 genome.

A nonreplicating plasmid (pLH02) was generated through two iterations of the Gibson assembly procedure (56) using a Gibson assembly master kit (New England BioLabs). Two iterations were used due to problems caused by high GC% in the *hmuR* gene (TM1040_0347) and earlier problems with assembly possibly due to the secondary structure of 5′ and 3′ overhangs of assembly fragments (see Fig. S4A in the supplemental material). The first-iteration plasmid (pLH01) was derived from pPY17a (26), a pRL271-derived suicide vector containing *sacB* for selection of double recombinants, by PCR amplification of two fragments with overlapping ends (gibfrag_01 and gibfrag_02) from plasmid pPY17a using primers gibfrag01_fwd and gibfrag01_rev and primers gibfrag02_fwd and and gibfrag02_rev. These fragments were combined with a portion of TM1040_0347 that was 645 bp long and 330 bp upstream of the 5′ start site. The TM1040_0347 fragment was amplified from TM1040 genomic DNA with primers (0347US_fwd and 0347US_rev) containing sequence overhangs matching gibfrag_01 and gibfrag_02. The three fragments were assembled through Gibson assembly, electroporated into *E. coli* DH5α, and then recovered by selection using kanamycin and sucrose sensitivity to isolate the correctly assembled pLH01. An analogous process was performed using pLH01 as a template to produce fragments gibfrag_03 and gibfrag_04 (using primers gibfrag03_fwd and gibfrag03_rev and primers gibfrag04_fwd and and gibfrag04_rev) and TM1040 genomic DNA to produce a 517-bp fragment 1,435 bp upstream of the 5′ start site of TM1040_0347 (using primers 0347DS_fwd and 0347DS_rev). These fragments were again assembled through Gibson assembly, electroporated into *E. coli* DH5α, and recovered by kanamycin selection and screening for sucrose sensitivity. The resulting plasmid (pLH02) contained a 1,162-bp portion of TM1040_0347 interrupted by a 460-bp deletion into which a kanamycin resistance cassette was inserted (Fig. S4A). All PCRs to produce Gibson assembly fragments were performed using Q5 High-Fidelity polymerase (NEB). Plasmids and bacterial strains used in this work are listed in Table S2 in the supplemental material.

The pLH02 plasmid was introduced into *Ruegeria* sp. strain TM1040 by electroporation as has been described earlier (42). Briefly, TM1040 was grown in 50 ml HISW medium at 30°C with shaking at 200 rpm to an OD_600_ of approximately 0.5, chilled on ice for 15 min, and centrifuged at 8,000 rpm for 10 min at 4°C. The supernatant was removed, and the cell pellet was gently resuspended in 10 ml of 10% glycerol–MQ water. The cell suspension was then centrifuged at 8,000 rpm for 10 min at 4°C, and the glycerol washing procedure was repeated for a total of three washes. After the final wash, the cell pellet was suspended in 0.5 ml of 10% glycerol–MQ water, a 65-μl aliquot was mixed with 50 ng of pLH02, and the mixture was incubated on ice for 1 min. Cells were electroporated in a 0.2-cm-path-length cuvette at 2,500 V, 400 Ω, and 25 μF and then immediately suspended in 1 ml of 30°C HISW medium and incubated at 30°C with shaking (200 rpm) for 2.5 h. Doubly recombinant mutants were selected on HISW plates containing 120 μg/ml kanamycin and 5% sucrose (wt/vol), and individual colonies were picked after approximately 14 h. TM1040 electroporated with plasmid pLH02 resulted in a recombinant strain (*ΔhmuR*975::*nptII*) with a partial deletion of *hmuR* (region 975 to 1,436 bp) and with an insertion of the neomycin phosphotransferase II gene (*nptII*), which confers kanamycin resistance. Transformants exhibiting both kanamycin resistance and no sucrose sensitivity were expected to have incorporated the mutagenic *ΔhmuR*975::*nptII* construct into the genome by double crossover (due to failed integration of the *sacB* gene, which confers sucrose sensitivity). The double-crossover event was verified by PCR to confirm correct insertion (using primers insrt_cnfirm_fwd and insrt_cnfirm_rev). A 2,926-bp insertion confirmed a 460-bp deletion in TM104_0347 and an insertion of the 1,374-bp neomycin phosphotransferase II gene in its place (Fig. S4B). The expected length of the PCR product of the wild-type gene using these primers is 2,013 bp.

### Preparation of trace-metal-clean algal lysate and removal of extracellular iron.

Axenic *Thalassiosira pseudonana* was grown in 1 liter of F/2-enriched seawater medium with 120 μM added EDTA until cells were at a density of 1 × 10^6^ to 2 × 10^6^ cells/ml. A 10-ml aliquot of the culture was filtered for chlorophyll *a* measurement, and the remaining volume was gently filtered (~5 mm Hg) through an acid-soaked 3.0-μm-pore-size Nuclepore filter, using an acid-cleaned Teflon filter rig. The cells were resuspended in an acid-cleaned centrifuge bottle in 200 ml of low-iron (~2 nM Fe) seawater collected from offshore California current water. A 20-ml volume of an oxalate wash solution (300 mM NaCl, 10 mM KCl, 100 mM Na_2_ oxalate, 50 mM Na_2_ EDTA [27]) was added to the resuspended cells in the low-iron seawater. The solution was mixed gently and allowed to rest at room temperature for 20 min, after which it was centrifuged at 6,000 rpm for 10 min at 4°C. The resulting cell pellet was washed again in low-iron seawater and oxalate solution for 20 min, centrifuged, and finally resuspended in 1 ml of trace metal clean Milli-Q water. A 300-μl volume of the suspension was immediately sonicated at 4°C using a Diogenode Bioruptor Standard system at the high-power setting for 6 min, using a 60-s on/30-s off cycle. The resulting soluble and insoluble lysate fractions were immediately frozen in liquid N_2_ until further use.

### Analytical measurement of heme *b* and chlorophyll from algal lysate.

Aliquots of algal lysate were mixed with two volumes of acidified acetone (80:20 [vol/vol] acetone/1.6 M HCl), ultrasonicated in a water bath sonicator (10 min at 4°C), and centrifuged at 13,000 rpm (10 min at 4°C) to extract heme *b* as described before (28, 57). The supernatant was diluted by a factor of 50 in mobile phase A, and heme *b* was measured spectrophotometrically by high-performance liquid chromatography (HPLC) using a polymeric reverse-phase column (PRP-1; Hamilton Inc.) (100 by 2.1 mm, 5-μm pore size) as described earlier (28, 58). HPLC was performed using binary gradient pumps (Waters 1525) with a microcell diode array spectrophotometer (Waters 2489) and manual full-loop injection (50 μl). Heme separation was completed at 22°C using a gradient of 100% A to 100% B over 10 min, followed by an isocratic elution of mobile phase B for 2 min at a flow rate of 0.5 ml/min. Mobile phases consisted of (i) 30:70:0.08 (vol/vol) acetonitrile/water/trifluoroacetic acid and (ii) 100:0.08 (vol/vol) acetonitrile/trifluoroacetic acid. Elution of heme *b* (retention time, 6.8 min) was monitored at 400 nm. For chlorophyll analysis, samples of *T. pseudonana* were filtered using 0.7-μm-pore-size GF/F filters (Whatman), stored in liquid N_2_ until use, extracted using a 90% acetone extraction/acidification method, and measured using a Turner Designs fluorometer (59).

### Study of competition between wild-type TM1040 and the LH02 mutant strain.

Quantitative PCR (qPCR) was performed on genomic DNA extracted from cocultures in order to calculate the abundances of TM1040 and LH02 strains. Oligonucleotide primers (Table S4) matching the neomycin phosphotransferase II gene in LH02 (kmR_F_compete and kmR_R_compete) and the 435-bp deleted portion of the TM1040 *hmuR* gene in LH02 (hmuR_F_compete and hmuR_R_compete) were used to distinguish between the TM1040 and LH02 strains. Five-point standard curves ranging from 311,268 to 31 genome copies per 25 μl of reaction mixture in 10-fold dilutions were generated for each primer pair, and reaction efficiencies were always between 95% and 100%. TM1040 and LH02 cultures were iron limited in PC+ marine medium with no added iron for two subsequent transfers and inoculated in duplicate into 20 ml PC+ medium at the same initial cell density (~5 × 10^6^ cells/ml). Cocultures were supplied with 500 nM FeCl_3_, 500 nM heme, or 10.6 nM heme equivalents of *Thalassiosira pseudonana* cellular lysate or with no additional iron source. Cocultures were maintained at 22°C with shaking at 190 rpm and were grown in the dark to prevent photodegradation of heme and hemoproteins. At each experimental time point, 2 ml of each coculture was pelleted by centrifugation and total DNA was extracted from cell pellets using a DNeasy blood and tissue kit (Qiagen) following the manufacturer’s protocols. Total genomic DNA from each coculture was quantified using a NanoDrop 1000-D Spectrophotometer (Nano-drop Technologies) and used in subsequent qPCR reactions.

10.1128/mSystems.00124-16.8 TABLE S4  Oligonucleotide primers used in this work. Letters in parentheses in the “Description” column indicate shorthand names for primers as designated in the supplemental figures. Download TABLE S4, DOCX file, 0.1 MB.. Copyright © 2017 Hogle et al. 2017 Hogle et al. This content is distributed under the terms of the Creative Commons Attribution 4.0 International license.

### Accession number(s).

Raw sequence data for reproducing all analyses can be obtained from the JGI IMG Integrated Microbial Genomes and Microbiomes database (see Data Set S1 for accession numbers) and the NCBI Gene Expression Omnibus (GEO GenBank accession number GSE65189) (Data Set S2). For user convenience, R scripts for processing transcriptomes and reproducing analysis are available from the Figshare repository at https://doi.org/10.6084/m9.figshare.4309547.

10.1128/mSystems.00124-16.10 DATA SET S2  IMG peptide accession numbers for TonB-dependent receptor sequence similarity network (Fig. 2). Download DATA SET S2, XLSX file, 0.03 MB.. Copyright © 2017 Hogle et al. 2017 Hogle et al. This content is distributed under the terms of the Creative Commons Attribution 4.0 International license.
