# Supplementary figures and images for: Direct Heme Uptake by Phytoplankton-Associated Roseobacter Bacteria
Source: mSystems. 2017 Jan 10;2(1):e00124-16. doi: 10.1128/mSystems.00124-16 (PMC5225302; doi:10.1128/mSystems.00124-16)

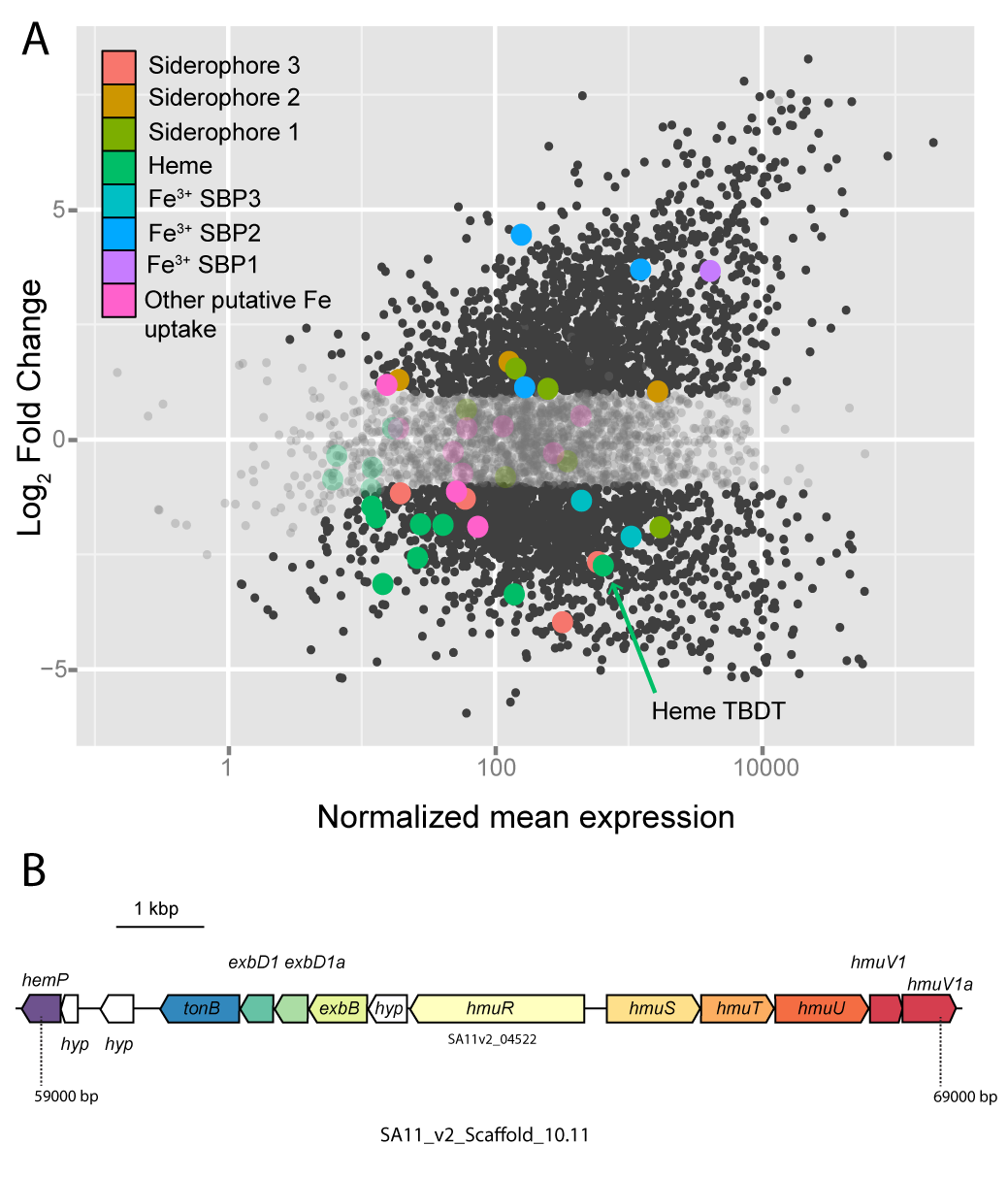

Supplement: FIG S1 [file sys001172079sf7.tif]

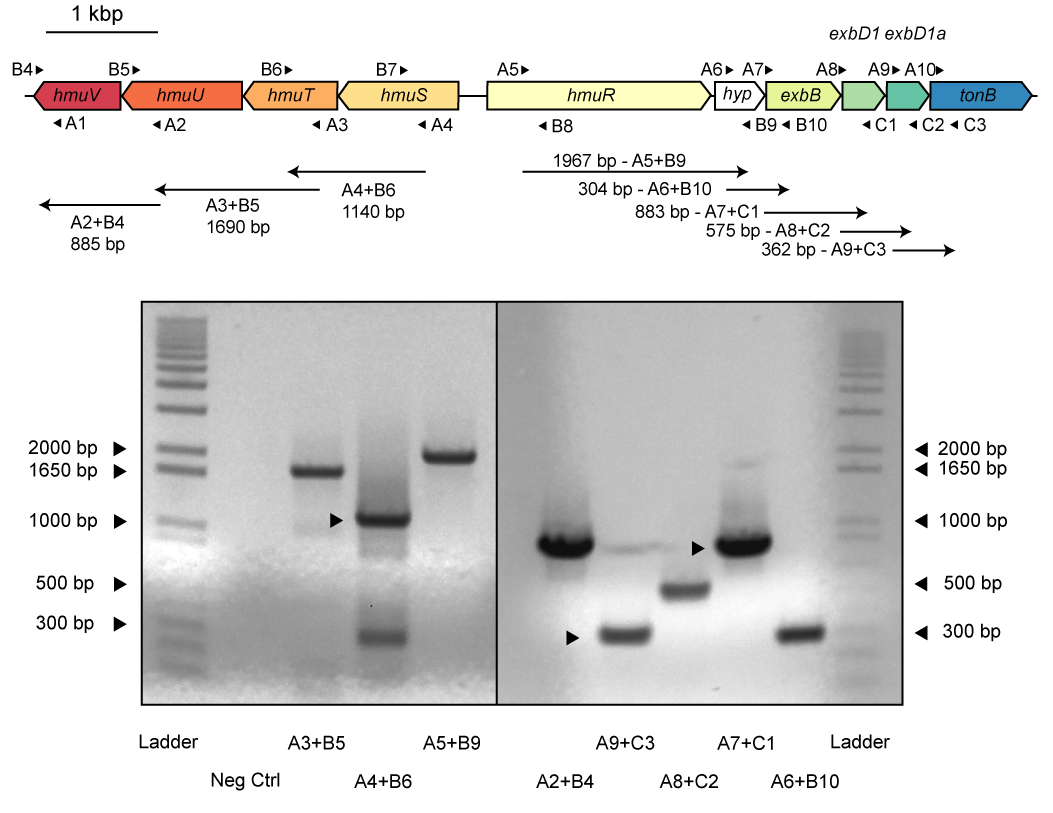

Supplement: FIG S2 [file sys001172079sf8.tif]

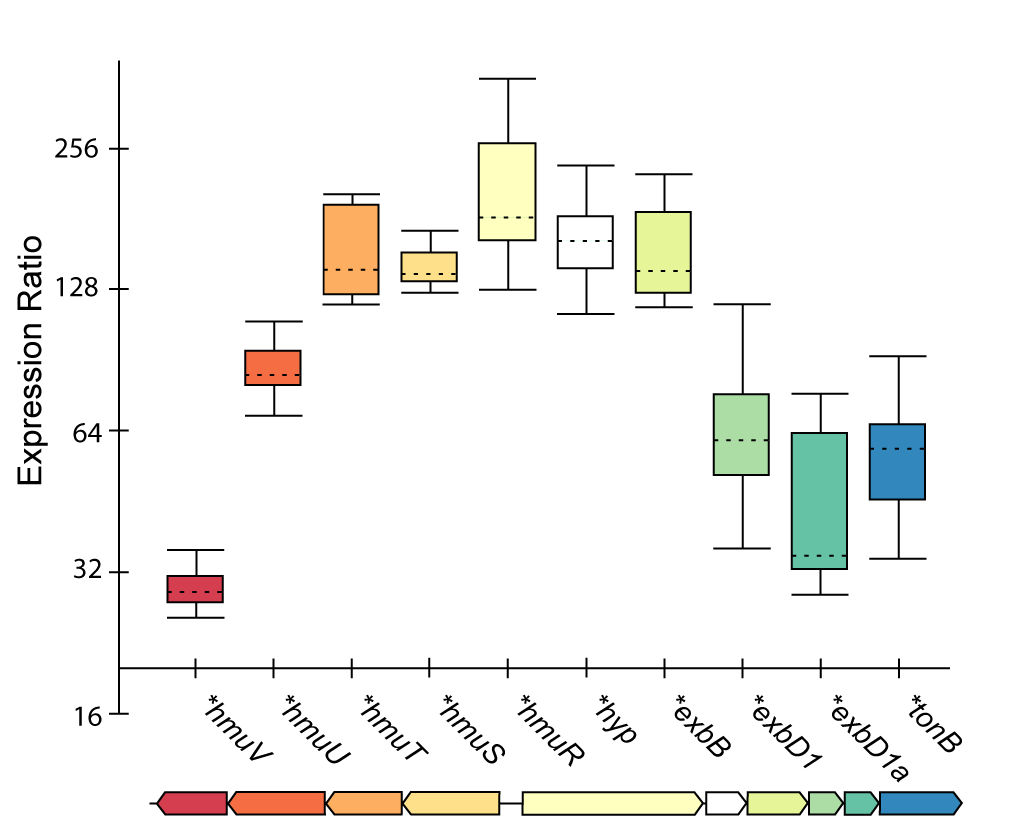

Supplement: FIG S3 [file sys001172079sf9.tif]

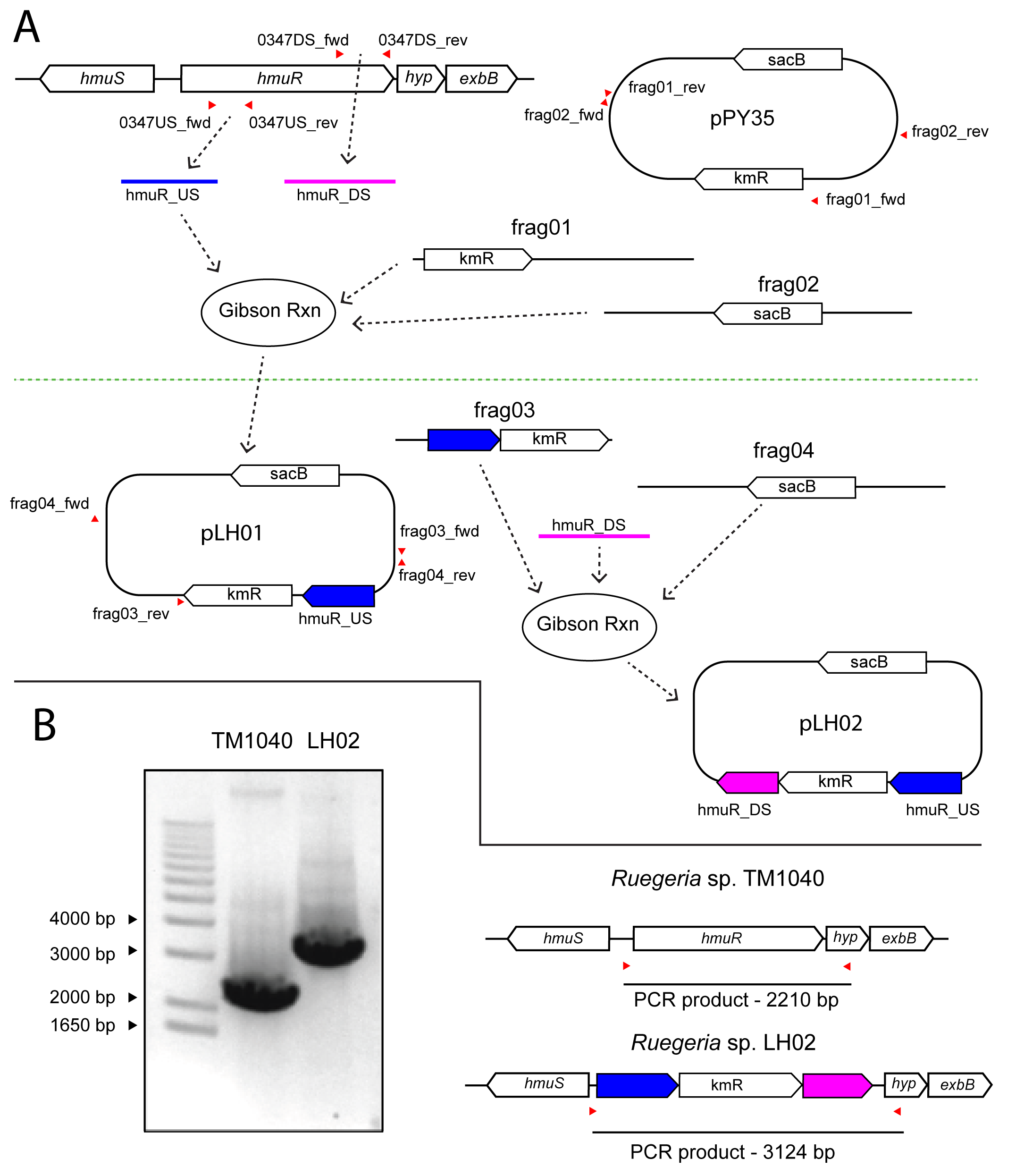

Supplement: FIG S4 [file sys001172079sf10.tif]
