# Supplementary material for: Direct Heme Uptake by Phytoplankton-Associated Roseobacter Bacteria
Source: mSystems. 2017 Jan 10;2(1):e00124-16. doi: 10.1128/mSystems.00124-16 (PMC5225302; doi:10.1128/mSystems.00124-16)
Supplement: TABLE S1 [file sys001172079st1.docx]

**Supplementary Table S1: Growth rates of TM1040 and LH02 on different Fe sources**

| Condition | Strain | Growth Rate (hr^-1^) | *P* value |
| --- | --- | --- | --- |
| 0 nM FeCl_3_ | TM1040 | 0.13 ± 0.004 | ns |
| 0 nM FeCl_3_ | LH02 | 0.13 ± 0.003 | ns |
| 500 nM FeCl_3_ | TM1040 | 0.31 ± 0.02 | ns |
| 500 nM FeCl_3_ | LH02 | 0.29 ± 0.02 | ns |
| 500 nM Heme | TM1040 | 0.28 ± 0.01 | < 0.0002 |
| 500 nM Heme | LH02 | 0.16 ± 0.01 | < 0.0002 |
| 17 μM Hemoglobin | TM1040 | 0.27 ± 0.006 | < 0.0002 |
| 17 μM Hemoglobin | LH02 | 0.20 ± 0.007 | < 0.0002 |
| 500 nM Cytochrome C | TM1040 | 0.14 ± 0.01 | ns |
| 500 nM Cytochrome C | LH02 | 0.15 ± 0.007 | ns |

*P* value indicates the results of an independent two tailed Student’s t test between strain growth rates at each condition. Values greater than 0.05 are omitted. There were three replicates for each condition.
