# Supplementary material for: Direct Heme Uptake by Phytoplankton-Associated Roseobacter Bacteria
Source: mSystems. 2017 Jan 10;2(1):e00124-16. doi: 10.1128/mSystems.00124-16 (PMC5225302; doi:10.1128/mSystems.00124-16)
Supplement: TABLE S2 [file sys001172079st2.docx]

**Supplementary Table S2: Bacterial strains and plasmids used in this work**

| Strain or Plasmid | Description^a,b^ | Source |
| --- | --- | --- |
| *E. coli* str. DH5α | Electrocompetent strain used as host for vector constructs. Catalog # C2989K | New England BioLabs |
| *Ruegeria* sp. TM1040 | *hmuR*+; wildtype strain | 1 |
| *Ruegeria* sp. LH02 | Δ*hmuR*975::*nptII*;  TM1040 electroporated with pLH02 resulting in a recombinant strain (LH02) with a partial deletion of *hmuR* (region 975-1436) and replacement of the neomycin phosphotransferase II gene which confers kanamycin resistance (Km^R^). | This work |
| pPY17a | Km^R^, Em^R^, Cm^R^, Suc^s^; used to construct pLH01 and pLH02 | 2 |
| pHL01 | Δ*hmuR*975::*nptII*; Suc^s^, used to construct pLH02 | This work |
| pHL02 | Δ*hmuR*975::*nptII;* Suc^s^, used for transformation to generate strain LH02. | This work |

1. Belas R, Horikawa E, Aizawa S-I, Suvanasuthi R (2009) Genetic determinants of Silicibacter sp. TM1040 motility. *J Bacteriol* 191(14):4502–4512.

2. Paz-Yepes J, Brahamsha B, Palenik B (2013) Role of a microcin-C-like biosynthetic gene cluster in allelopathic interactions in marine *Synechococcus*. *Proc Natl Acad Sci U S A* 110(29):12030–12035.

^a^Km^R^, Em^R^, Cm^R^ indicate resistance to kanamycin, erythromycin, and chloramphenicol

^b^Suc^S^ indicates growth sensitivity to 5% wt/vol sucrose as conferred by the *sacB* gene
