# Supplementary material for: Direct Heme Uptake by Phytoplankton-Associated Roseobacter Bacteria
Source: mSystems. 2017 Jan 10;2(1):e00124-16. doi: 10.1128/mSystems.00124-16 (PMC5225302; doi:10.1128/mSystems.00124-16)
Supplement: TABLE S3 [file sys001172079st3.docx]

**Supplementary Table S3: Measurements of heme content in soluble and insoluble *T. pseudonana* lysate**

| Culture | heme *b* in lysate (nM) | heme *b* in original culture (pM) | Chl *a* (nM) | Cells L^-1^ | heme *b* (nmol) cell^-1^ | Chl *a* (nmol) cell^-1^ | Chl *a* : heme *b* |
| --- | --- | --- | --- | --- | --- | --- | --- |
| *T. pseudonana* 3 | 761 | 684 | 340 | ^a^1.24 ✕ 10^6^  ^b^7.67 ✕ 10^8^ | 9.92 ✕ 10^-7^ | 2.74 ✕ 10^-4^ | 276.45 |
| *T. pseudonana* 2 | 831 | 748 | 342 | ^a^1.30 ✕ 10^6^  ^b^7.00 ✕ 10^8^ | 1.19 ✕ 10^-6^ | 2.63 ✕ 10^-4^ | 221.54 |
| *T. pseudonana* 1 | 624 | 562 | 335 | ^a^1.10 ✕ 10^6^  ^b^6.05 ✕ 10^8^ | 1.03 ✕ 10^-6^ | 3.05 ✕ 10^-4^ | 295.03 |

*^a^*Cell concentration before washing and concentration step. Used to calculate Chl *a* cell^-1^

*^b^*Cell concentration after washing and concentration step. Used to calculate heme *b* cell^-1^
