# Supplementary material for: Direct Heme Uptake by Phytoplankton-Associated Roseobacter Bacteria
Source: mSystems. 2017 Jan 10;2(1):e00124-16. doi: 10.1128/mSystems.00124-16 (PMC5225302; doi:10.1128/mSystems.00124-16)
Supplement: TABLE S4 [file sys001172079st4.docx]

**Supplementary Table S4: Oligonucleotide primers used in this work**

| Primer name | Sequence (5' to 3') | Description |
| --- | --- | --- |
| gibfrag01_fwd | ACACAAAAGAACCCCCAGA | Amplify fragment 01 from pPY17a for first Gibson construction |
| gibfrag01_rev | CAGGAAAGAACATGTGAGCAAA | Amplify fragment 01 from pPY17a for first Gibson construction |
| gibfrag02_fwd | CCTTTTGCTGGCCTTTTG | Amplify fragment 02 from pPY17a for first Gibson construction |
| gibfrag02_rev | GTTAAGGGATGCAGGTCG | Amplify fragment 02 from pPY17a for first Gibson construction |
| 0347US_fwd | CGACCTGCATCCCTTAACAACCAAAAGGTCCAGATCC | Amplify upstream region of *hmuR* from TM1040 genomic DNA and add overhangs. Used in first Gibson construction |
| 0347US_rev | TCTGGGGGTTCTTTTGTGTCTCGATGTATTGATCCGCAT | Amplify upstream region of *hmuR* from TM1040 genomic DNA and add overhangs. Used in first Gibson construction |
| gibfrag03_fwd | AAATAATAGTGAACGGCAGG | Amplify fragment 03 from pLH01 for second Gibson construction |
| gibfrag03_rev | AGAGCTTTGTTGTAGGTG | Amplify fragment 03 from pLH01 for second Gibson construction |
| gibfrag04_fwd | TACGATACACTTCCGCTC | Amplify fragment 04 from pLH01 for second Gibson construction |
| gibfrag04_rev | ACCCATCACATATACCTG | Amplify fragment 04 from pLH01 for second Gibson construction |
| 0347DS_fwd | CACCTACAACAAAGCTCTCCCAGACTGAAAAAGCGG | Amplify downstream region of *hmuR* from TM1040 genomic DNA and add overhangs. Used in second Gibson construction |
| 0347DS_rev | GAGCGGAAGTGTATCGTATTGCGTCTGATATTGCTTGT | Amplify downstream region of *hmuR* from TM1040 genomic DNA and add overhangs. Used in second Gibson construction |
| insrt_cnfirm_fwd | AGAGGCCGCAAGAGTGAA | Used to confirm double cross-over event in R. LH02 |
| insrt_cnfirm_rev | GAGGGAGGTGAGGACAGCAA | Used to confirm double cross-over event in R. LH02 |
| hmuV_F | ATCAACGCCTGTCAGAGGTC | (B4) – used in RT-qPCR |
| hmuV_R | GTTTGCGGCAGAACAAAGAC | (A1) – used in RT-qPCR |
| hmuU_F | TCATCAGCCGTACCATCATC | (B5) – used in RT-qPCR |
| hmuU_R | CGTTTCAGCAAGATCCACAG | (A2) – used in RT-qPCR |
| hmuT_F | TATCCGTGAACCCAAACCTG | (B6) – used in RT-qPCR |
| hmuT_R | GTTGATCTTGTCGACGATGC | (A3) – used in RT-qPCR |
| hmuS_F | GATTCTGCAGCCAGAAATCG | (B7) – used in RT-qPCR |
| hmuS_R | TGTCGGTGTCTTCATTCAGG | (A4) – used in RT-qPCR |
| hmuR_F | AATTGACGACCGATCCTGAG | (A5) – used in RT-qPCR |
| hmuR_R | CGGAGCCATATTCAAACGAC | (B8) – used in RT-qPCR |
| hyp_F | GATGCTTCTGACGCTCTTTG | (A6) – used in RT-qPCR |
| hyp_R | ACACCAGCAACATCGAACTG | (B9) – used in RT-qPCR |
| exbB_F | GTTCTGGACGGCAATAAAGG | (A7) – used in RT-qPCR |
| exbB_R | CGATCGACAGTCCAATAAGC | (B10) – used in RT-qPCR |
| exbD1_F | TGCTCTCTTCGACGTTTTCG | (A8) – used in RT-qPCR |
| exbD1_R | CTTCACCATTCAGCGACATC | (C1) – used in RT-qPCR |
| exbD1a_F | GATGGTAGCCTGCATTTTCG | (A9) – used in RT-qPCR |
| exbD1a_R | TTCCTCTTCCCAGATGATGC | (C2) – used in RT-qPCR |
| tonB_F | AAGAGACCGCTGTGGAAATC | (A10) – used in RT-qPCR |
| tonB_R | CTGAACAAGGTCCGCAAAAC | (C3) – used in RT-qPCR |
| rpoD_F | CGCCAAGAAATACACCAACC | - used in RT-qPCR |
| rpoD_R | TTATAGCCGCGACGGTATTC | - used in RT-qPCR |
| gyrA_F | ACGCTCTTTGTGGCGAATAC | - used in RT-qPCR |
| gyrA_R | GGGATTGGCAGAATGTTGAC | - used in RT-qPCR |
| gmkA_F | TGCTCTTTGACATCGACTGG | - used in RT-qPCR |
| gmkA_R | AGATCGACAGCGTGTGTTTG | - used in RT-qPCR |
| kmR_F_compete | ATTCTCACCGGATTCAGTCG | - used for TM1040 + LH02 competition exp |
| kmR_R_compete | ATTCCGACTCGTCCAACATC | - used for TM1040 + LH02 competition exp |
| hmuR_F_compete | AAAGGAACGGTTGAATGCGG | - used for TM1040 + LH02 competition exp |
| hmuR_R_compete | GATCTCCTGGCGTTCGTAGC | - used for TM1040 + LH02 competition exp |

Letters in parentheses in the “Description” column indicate shorthand names for primers as designated in the figures
